# *Calycomorphotria hydatis* gen. nov., sp. nov., a novel species in the family *Planctomycetaceae* with conspicuous subcellular structures

**DOI:** 10.1007/s10482-020-01419-0

**Published:** 2020-05-12

**Authors:** Torsten Schubert, Nicolai Kallscheuer, Sandra Wiegand, Christian Boedeker, Stijn H. Peeters, Mareike Jogler, Anja Heuer, Mike S. M. Jetten, Manfred Rohde, Christian Jogler

**Affiliations:** 1 Department of Microbial Interactions, Institute of Microbiology, Friedrich Schiller University, Jena, Germany; 2 Department of Microbiology, Radboud University, Nijmegen, The Netherlands; 3 Institute for Biological Interfaces 5, Karlsruhe Institute of Technology, Eggenstein-Leopoldshafen, Germany; 4 Leibniz Institute DSMZ, Braunschweig, Germany; 5 Central Facility for Microscopy, Helmholtz Centre for Infection Research, Braunschweig, Germany

**Keywords:** Marine bacteria, *Planctomycetes*, Cell biology, Membrane invaginations, Aggregation

## Abstract

A novel strain belonging to the family *Planctomycetaceae*, designated V22^T^, was isolated from sediment of a seawater fish tank in Braunschweig, Germany. The isolate forms pink colonies on solid medium and displays common characteristics of planctomycetal strains, such as division by budding, formation of rosettes, a condensed nucleoid and presence of crateriform structures and fimbriae. Unusual invaginations of the cytoplasmic membrane and filamentous putative cytoskeletal elements were observed in thin sections analysed by transmission electron microscopy. Strain V22^T^ is an aerobic heterotroph showing optimal growth at 30 °C and pH 8.5. During laboratory cultivations, strain V22^T^ reached generation times of 10 h (maximal growth rate of 0.069 h^−1^). Its genome has a size of 5.2 Mb and a G + C content of 54.9%. Phylogenetically, the strain represents a novel genus and species in the family *Planctomycetaceae*, order *Planctomycetales*, class *Planctomycetia*. We propose the name *Calycomorphotria hydatis* gen. nov., sp. nov. for the novel taxon, represented by the type strain V22^T^ (DSM 29767^T^ = LMG 29080^T^).

## Introduction

*Planctomycetes*, an ubiquitous phylum of bacteria of mostly aquatic origin (Wiegand et al. 2018), comprises species with an uncommon physiology and morphology among bacteria (Lage et al. 2019; Wiegand et al. 2020). Phylogenetically, the phylum *Planctomycetes*, together with *Chlamydiae*, *Verrucomicrobia* and others, forms the PVC superphylum (van Niftrik and Devos 2017). The phylum *Planctomycetes* is subdivided into the classes *Candidatus* Brocadiae, *Phycisphaerae* and *Planctomycetia.* The taxonomy of the family *Planctomycetia* was recently revised, which led to further subdivision into the orders *Gemmatales*, *Isosphaerales*, *Pirellulales* and *Planctomycetales* (Dedysh et al. 2019b)*.* Species of *Candidatus* Brocadiae are capable of performing anaerobic ammonium oxidation (anammox) (Strous et al. 1999) and thereby convert ammonium to dinitrogen gas (Peeters and van Niftrik 2018). Members of the class *Planctomycetia* have been isolated from various aquatic biotic and abiotic surfaces in the past decade (Boersma et al. 2019; Bondoso et al. 2014, 2017; Kallscheuer et al. 2020; Peeters et al. 2020; Vollmers et al. 2017). Such species can be highly abundant on marine biotic surfaces, e.g. on macroscopic phototrophs (Bengtsson and Øvreås 2010). Since oceans are typically oligotrophic, species of the class *Planctomycetia* probably digest complex carbon substrates derived from the biotic surfaces to which they are attached (Jeske et al. 2013; Lachnit et al. 2013). For this purpose, they may utilise a specialised machinery for the uptake and intracellular digestion of complex polysaccharides (Boedeker et al. 2017), which could be a decisive advantage during competition for nutrients in their natural habitats.

In recent years, microscopic techniques and genetic tools (Jogler et al. 2011; Jogler and Jogler 2013; Rivas-Marin et al. 2016) have enabled a detailed analysis of the cell envelope architecture of Planctomycetes. Both, *Planctomycetes* and *Verrucomicrobia*, were shown to possess peptidoglycan (Jeske et al. 2015; Rast et al. 2017; van Teeseling et al. 2015). The cell envelope architecture of Planctomycetes is therefore similar to that of Gram-negative bacteria (Boedeker et al. 2017; Devos 2014a, b). Nevertheless, Planctomycetes display uncommon cell biological features, e.g. with regard to their mode of cell division. Members of the class *Planctomycetia* divide by budding, whereas *Phycisphaerae* divide by binary fission. Some species probably even switch between both modes of division (Wiegand et al. 2020). All characterised members of the phylum lack canonical divisome proteins including the otherwise universal FtsZ (Jogler et al. 2012; Pilhofer et al. 2008). The sizes of planctomycetal genomes range between 3 and 12 Mb (Ravin et al. 2018; Wiegand et al. 2018), while typically 40–55% of the gene products are annotated as hypothetical or uncharacterised proteins. Given such values, Planctomycetes represent an attractive subject for future research.

Here, we describe a novel strain, V22^T^, isolated from sediment of a seawater fish tank in Braunschweig, Germany. According to the results of our analysis, the strain represents a novel species and genus in the recently revisited family *Planctomycetaceae*, order *Planctomycetales* in the class *Planctomycetia* (Dedysh et al. 2019b).

## Material and methods

### Isolation of the novel strain and cultivation

For the isolation and cultivation of strain V22^T^, M1H NAG ASW medium was prepared as described by Boersma et al. (2019). Sediment and water from a seawater fish tank in Braunschweig, Germany (location: 52.2689 N 10.5268 E) were mixed by shaking and the water was subsequently plated on M1H NAG ASW plates containing 8 g/L gellan gum, 1000 mg/L streptomycin, 200 mg/L ampicillin and 20 mg/L cycloheximide, which were then incubated at 20 °C for several weeks. In order to verify that obtained strains are members of the phylum *Planctomycetes*, the 16S rRNA gene was amplified using colony-PCR and subsequently sequenced as previously described (Rast et al. 2017).

### Determination of pH and temperature optimum

Cultivations for determination of the pH optimum were performed in M1H NAG ASW medium. A concentration of 100 mM 4-(2-hydroxyethyl)-1-piperazineethanesulfonic acid (HEPES) was used for cultivations at pH 7, 7.5 and 8. For cultivation at pH 5 and 6, HEPES was replaced by 100 mM 2-(*N*-morpholino)ethanesulfonic acid (MES), whereas 100 mM *N*-cyclohexyl-2-aminoethanesulfonic acid (CHES) served as a buffering agent at pH 9 and 10. Cultivations for determination of the pH optimum were performed at 28 °C. Cultivations for determination of the temperature optimum were performed in standard M1H NAG ASW medium at pH 7.5.

### Microscopy protocols

Phase contrast and field emission scanning electron microscopy were performed as previously described (Boersma et al. 2019). Thin sectioning, subsequent staining and transmission electron microscopy (TEM) were performed as previously described (Jogler et al. 2011).

### Genome information

The *g*enome sequence of strain V22^T^ is available from GenBank under accession number CP036316. The 16S rRNA gene sequence of strain V22^T^ can be found under accession number MK554537. Sequencing of the genome of strain V22^T^ is described in a previously published study (Wiegand et al. 2020). Numbers of carbohydrate-active enzymes were obtained from the CAZY database (Lombard et al. 2014). Gene clusters potentially involved in the production of secondary metabolites were determined using antiSMASH 4.0 (Blin et al. 2017).

### Phylogenetic analysis

16S rRNA gene sequence-based phylogeny was computed for strain V22^T^, the type strains of all described planctomycetal species (assessed in January 2020) and all isolates recently published and described (Boersma et al. 2019; Dedysh et al. 2019a, b; Kallscheuer et al. 2019a, b, 2020; Kohn et al. 2019; Peeters et al. 2020; Rensink et al. 2020). The 16S rRNA gene sequences were aligned with SINA (Pruesse et al. 2012) and the phylogenetic inference was calculated with RAxML (Stamatakis 2014) with a maximum likelihood approach with 1000 bootstraps, nucleotide substitution model GTR, gamma distributed rate variation and estimation of proportion of invariable sites (GTRGAMMAI option). For the multi-locus sequence analysis (MLSA) the unique single-copy core genome of the analysed genomes was determined with proteinortho5 (Lechner et al. 2011) with the ‘selfblast’ option enabled. The protein sequences of the resulting orthologous groups were aligned using MUSCLE v.3.8.31 (Edgar 2004). After clipping, partially aligned *C*- and *N*-terminal regions and poorly aligned internal regions were filtered using Gblocks (Castresana 2000). The final alignment was concatenated and clustered using FastTree (Price et al. 2009). The average nucleotide identity (ANI) was calculated using OrthoANI (Lee et al. 2016). The average amino acid identity (AAI) was calculated using the aai.rb script of the enveomics collection (Rodriguez-R and Konstantinidis 2016) and the percentage of conserved proteins (POCP) was calculated as described (Qin et al. 2014). The *rpoB* nucleotide sequences were taken from publicly available planctomycetal genome annotations and the sequence identities were determined as described (Bondoso et al. 2013). Upon extracting only those parts of the sequence that would have been sequenced with the described primer set, the alignment and matrix calculation was performed with Clustal Omega (Sievers et al. 2011).

## Results and discussion

### Phylogenetic inference

**Fig. 1:**
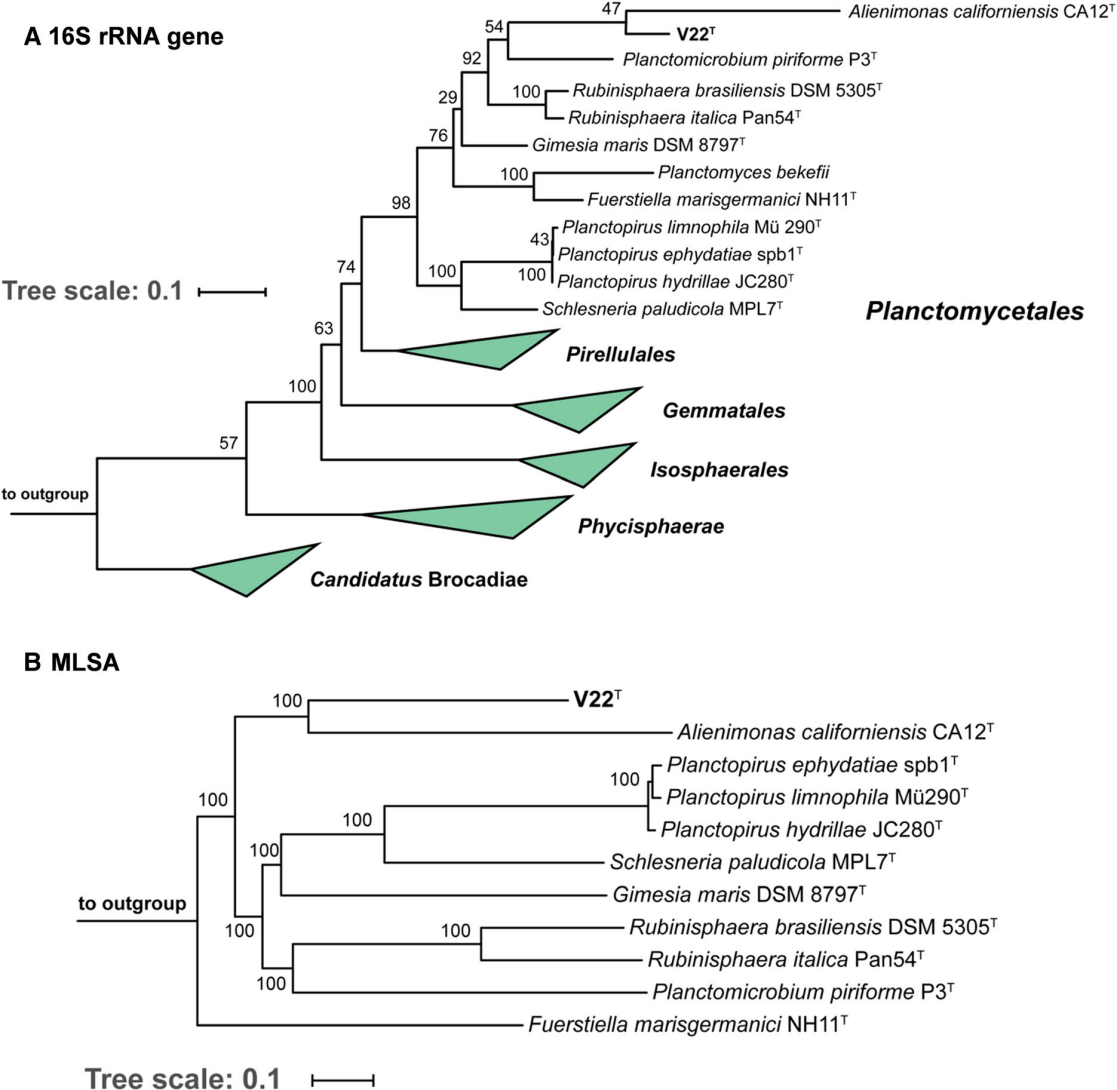
Maximum likelihood phylogenetic analysis. Phylogenetic tree showing the position of strain V22^T^. 16S rRNA gene sequence- and MLSA-based phylogeny was computed as described in the Material and Methods section. Bootstrap values after 1,000 re-samplings (16S rRNA-based tree) are given at the nodes (in %). The outgroup consists of three 16S rRNA genes from the PVC superphylum outside of the phylum *Planctomycetes* (GenBank accession numbers AJ229235, NR_146840 and NR_027571). In the MLSA-based tree confidence values are given at the nodes (in %). Six species of the family *Pirellulaceae* served as outgroup

**Fig. 2:**
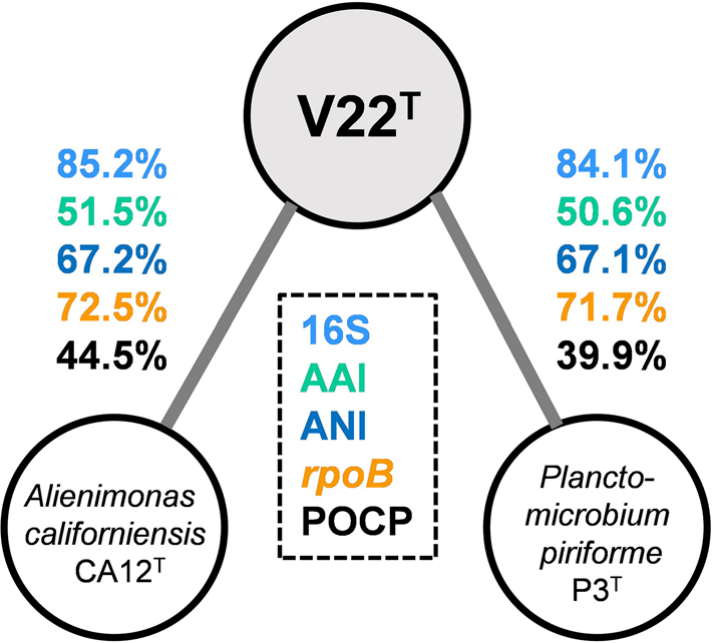
Phylogenetic marker values of strain V22^T^ and its currently closest neighbours. The numbers give the similarity values shared between strain V22^T^, *Alienimonas californiensis* CA12^T^ and *Planctomicrobium pirifome* P3^T^ for 16S rRNA gene sequence identity (16S), average amino acid identity (AAI), average nucleotide identity (ANI), *rpoB* nucleotide sequence identity (*rpoB*) and percentage of conserved proteins (POCP)

Based on 16S rRNA gene phylogeny (Fig. 1a), strain V22^T^ belongs to the recently redefined family *Planctomycetaceae,* the sole family in the order *Planctomycetales* (Dedysh et al. 2019b). This family is currently formed by eight genera, namely *Gimesia*, *Planctomyces*, *Planctopirus*, *Schlesneria*, *Planctomicrobium*, *Rubinisphaera*, *Fuerstiella* and *Alienimonas*. Within this family, the current closest relative of strain V22^T^ is *Alienimonas californiensis* CA12^T^ (Boersma et al. 2019) as determined by both 16S rRNA gene analysis and MLSA (Fig. 1b). Strain V22^T^ shares a 16S rRNA gene identity of 85.2% with *A. californiensis*, a value clearly below the genus threshold of 94.5% and even slightly below the family threshold of 86.5% (Yarza et al. 2014). This strongly supports delineation of strain V22^T^ from the genus *Alienimonas*. In order to substantiate the delineation from known genera additional phylogenetic markers were taken into consideration, e.g. AAI, *rpoB* similarity and POCP. Similarity values obtained during comparison of strain V22^T^ with *A. californiensis* and *Planctomicrobium piriforme*, a second close relative, are below the genus threshold values applied for *rpoB* (75.5–78.0%) (Kallscheuer et al. 2019b), AAI (60–80%) (Konstantinidis and Tiedje 2005), and POCP (50%) (Qin et al. 2014) (Fig. 2). ANI values of 67.2% and 67.1% confirm that strain V22^T^ does not belong to *A. californiensis* or *P. piriforme*. Hence, all used methods support the delineation of strain V22^T^ from members of already described genera in the family *Planctomycetaceae*.

### Morphological, physiological and biochemical analyses

**Fig. 3:**
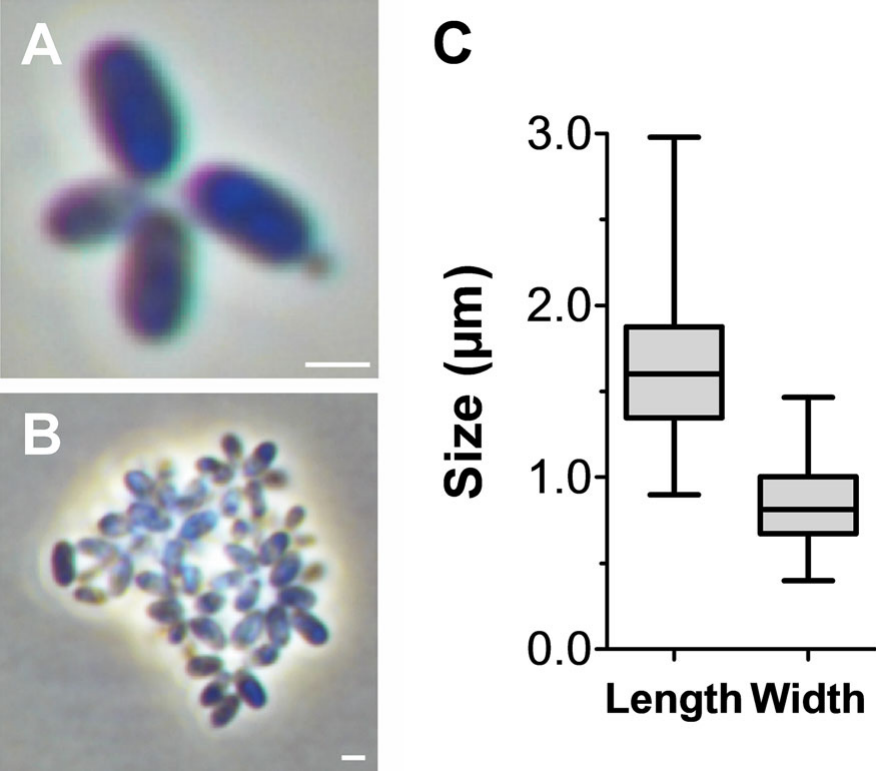
Light microscopy images and cell size plot of strain V22^T^. The mode of cell division (A) and a general overview of cell morphology (B) is shown in the pictures. The scale bar is 1 µm. For determination of the cell size (C) at least 100 representative cells were counted manually or by using a semi-automated object count tool

**Fig. 4:**
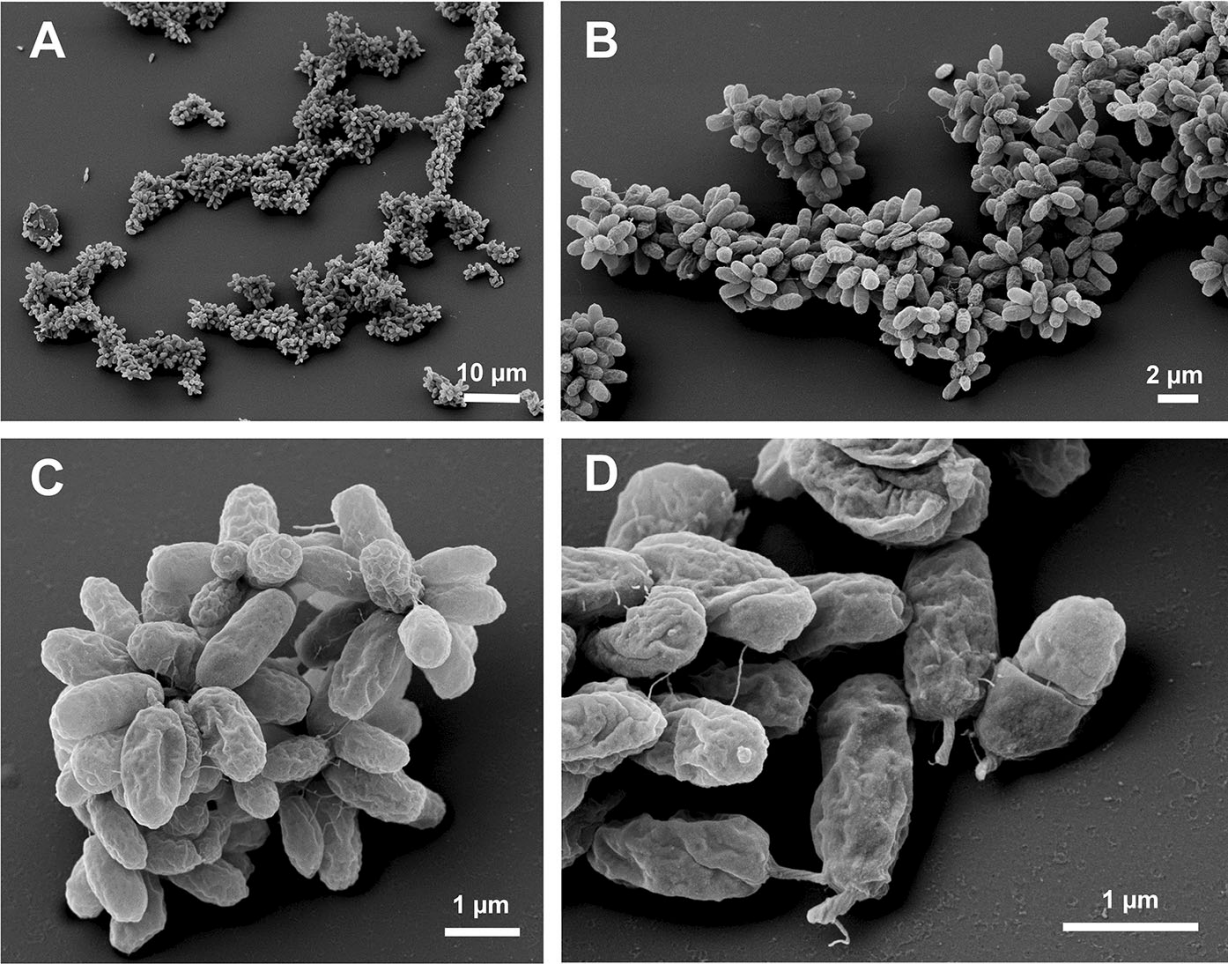
Electron microscopic images of strain V22^T^. Cells of strain V22^T^ form rosettes typically comprising 6–15 cells. The rosettes assemble to linear or slightly branched chains. Separate scale bars are presented in each of the images

**Fig. 5:**
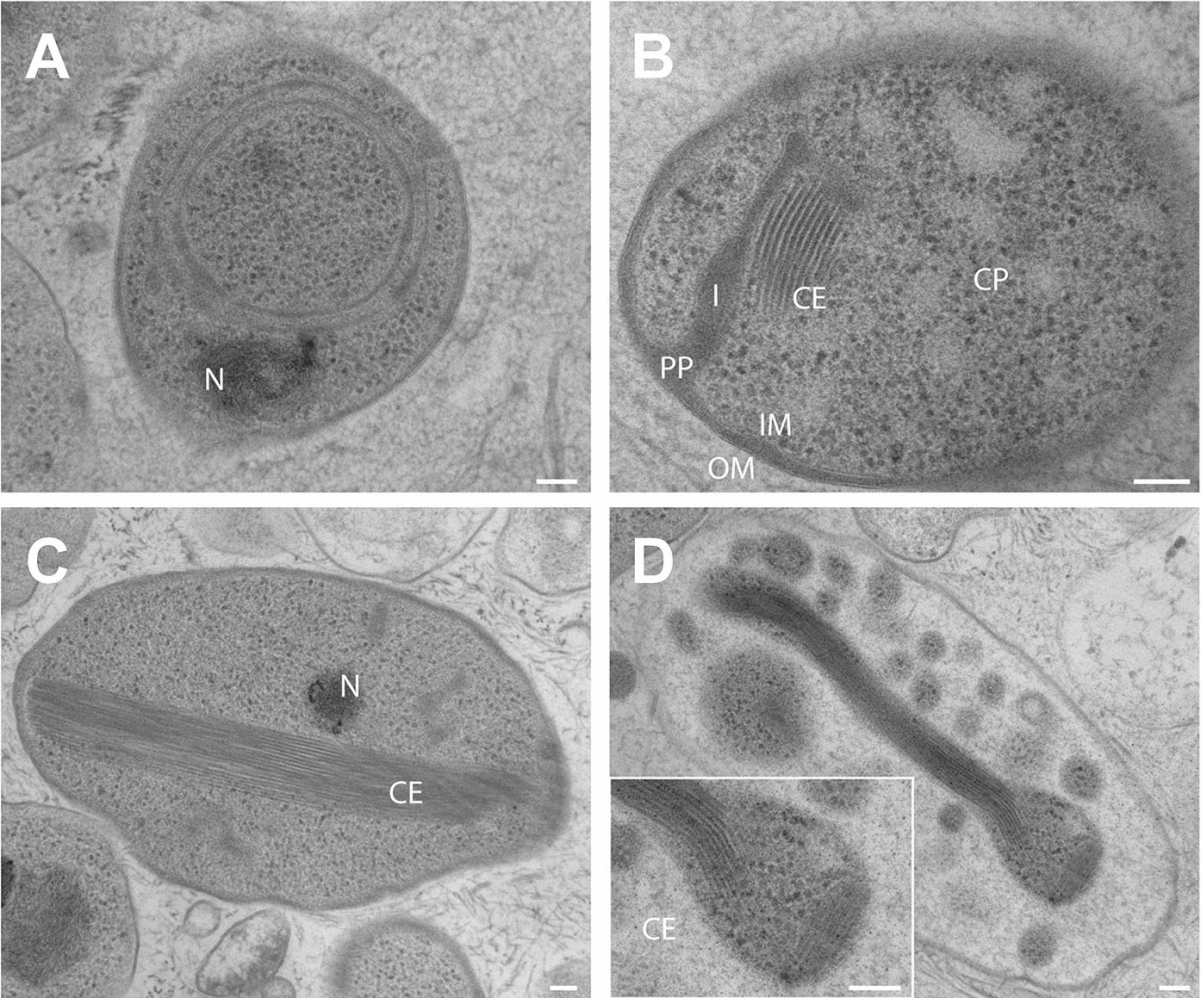
Transmission electron microscopy images of strain V22^T^. TEM images of thin sections showing the cell morphology of strain V22^T^. Abbreviations: CE: (potential) cytoskeletal elements, CP cytoplasm, I invagination, IM inner membrane, OM outer membrane, PP periplasmic space, N condensed nucleoid. The scale bar is 0.1 µm

Basic features of strain V22^T^ including cell morphology, growth and mechanism of cell division are summarised in Table 1 and compared to the currently closest neighbours *A. californiensis* and *P. piriforme*. Exponentially growing V22^T^ cells were analysed using phase contrast, SEM and TEM analysis. Cells of strain V22^T^ are round grain rice-shaped with an average size of 1.6 ± 0.3 µm (length) and 0.7 ± 0.2 µm (width) (Fig. 3a, c). Cells of *A. californiensis* CA12^T^ and *P. piriforme* P3^T^ are larger than V22^T^ cells. Strain V22^T^ forms rosettes of typically 6–15 cells, which assemble to characteristic linear or slightly branched chains (Fig. 4). Crateriform structures are present on the surface of all three strains, but their distribution is different (Table 1). TEM thin sections of strain V22^T^ confirm a planctomycetal cell architecture with a condensed nucleoid and invaginations of the cytoplasmic membrane leading to an enlarged periplasmic space (Fig. 5). In the cytoplasm of strain V22^T^, cytoskeletal elements (CE) were observed (Fig. 5b–d). Such putative CEs form partly thick bundles (Fig. 5c), which seem to fill membrane cavities (Fig. 5d). The presence of such structures has been reported in Planctomycetes before (Lage et al. 2013).

**Table 1:** Phenotypic and genotypic features of strain V22^T^ compared to closely related strains

Feature	V22^T^	*A. californiensis* CA12^T^	*P. piriforme* P3^T^
Phenotypic characteristics			
Shape	Round grain rice-shaped	Spherical to ovoid	Ellipsoid to pear-shaped
Length [µm]	1.6 ± 0.3	2.0 ± 0.2	1.7–2.8
Width [µm]	0.7 ± 0.2	1.5 ± 0.3	0.9–1.3
Colour	Pink	Pink	White
Relation to oxygen	Aerobic	Aerobic	Aerobic
Temperature range (optimum) [°C]	15–32 (30)	10–36 (27)	10–30 (20–28)
pH range (optimum)	6.0–10.0 (8.5)	6.0–9.0 (7.5)	4.2–7.1 (6.0–6.5)
Division	Budding	Budding	Budding
Dimorphic life cycle	No	No	Yes
Motility	Yes	Yes	Yes
Crateriform structures	Overall	Overall, except for the pole at which the flagellum is located	At reproductive pole
Fimbriae	Few	Yes, polar	n.d.
Capsule	n.o.	n.o.	n.d.
Stalk	Short, opposite of budding pole	n.o.	Yes
Holdfast structure	n.o.	n.o.	n.d.
Genomic characteristics			
Genome size [bp]	5,163,473	5,475,215	6,317,004
G + C [%]	53.9	70.7	58.8
Coding density [%]	87.8	88.5	85.8
Completeness [%]	94.8	94.8	95.7
Contamination [%]	0	0	1.72
Total genes	4,376	4,382	5,117
Protein-coding genes	4,301	4,309	5,050
Hypothetical proteins	1,673	1,798	2,814
16S rRNA genes	2	2	1
tRNA genes	64	65	53

The genome analysis is based on GenBank accession numbers CP036316 (V22^T^), CP036265 (*Alienimonas californiensis* CA12^T^) and GCA_900113665 (*Planctomicrobium piriforme* P3^T^)

*n.o.* not observed, *n.d*. not determined

The three compared strains are aerobic and motile. Strain V22^T^ shares pink pigmentation with *A. californiensis*, whereas *P. piriforme* is non-pigmented. In M1H NAG ASW medium, strain V22^T^ was able to growth over a temperature range of 15–32 °C and a pH range of 6.0–10.0. Strain V22^T^ is heterotrophic with a mesophilic and slightly alkaliphilic growth profile. The new isolate originated from sediment of a seawater fish tank, which might indicate a facultatively anaerobic lifestyle. Whether growth under oxygen depletion is based on anaerobic respiration or fermentation needs further studies. The presence of menaquinone biosynthesis genes has been verified in the genome sequence, however, the production of menaquinones is not indicative of an anaerobic energy metabolism. *Planctomycetales* generally produce menaquinones (Sittig and Schlesner 1993). Strain V22^T^ harbours the gene for a cobalamin-containing methionine synthase (*metH*, V22_33860). Since an open reading frame encoding the cobalamin-independent variant of the essential anabolic enzyme has not been identified, the organism might be a cobalamin auxotroph that salvages B_12_ vitamins from the environment.

Optimal growth of strain V22^T^ was observed at 30 °C and pH 8.5 (Table 1), which led to a maximal growth rate of 0.069 h^−1^, corresponding to a generation time of 10 h. While the pH range allowing growth of strain V22^T^ is similar to that of *A. californiensis*, *P. piriforme* prefers slightly acidic conditions. *A. californiensis* can grow up to temperatures of 36 °C, while the other two strains only grew up to 30–32 °C. Taken together, the three strains can be clearly differentiated using morphological and physiological properties.

### Genomic characteristics and genome-based analysis of metabolic capabilities

The genome of strain V22^T^ has a size of 5.2 Mb and a G + C content of 53.9%. The genome of strain V22^T^ and *A. californiensis* have a comparable size, whereas the genome of *P. piriforme* is 1 Mb larger. The numbers of tRNA genes in the three strains range between 53–65. Planctomycetal genomes typically contain a high percentage of genes coding for hypothetical or uncharacterised proteins. The values are in a comparable range (39–42%) in case of strain V22^T^ and *A. californiensis*, but lower than the 56% in *P. piriforme*. Hence, the latter has about 700 hypothetical proteins more than the other two strains. *A. californiensis* has a high G + C content of 71%, which is a distinctive criterion for delineation from strain V22^T^ and *P. piriforme*, which have a moderate G + C content of 54–59%.

Based on the genome sequences of strain V22^T^ and the two current close neighbours, we analysed the numbers of genes coding for enzymes putatively involved in the degradation of polysaccharides (carbohydrate-active enzymes) or in biosynthetic pathways for secondary metabolites (Table 2). Carbohydrate-active enzymes could not be analyzed for *P. piriforme* as the CAZY database only lists strains with complete genomes. Strain V22^T^ and *A. californiensis* CA12^T^ harbour 96–122 carbohydrate-active enzymes and show a similar distribution pattern with regard to the different families. Approximately 60% of the identified enzymes could be assigned to the glycosyl transferase family in both strains. Numbers in the other families vary only slightly between the two strains. Numbers of predicted clusters involved in the secondary metabolism of strain V22^T^ are surprisingly low. The strain harbours only three terpenoid biosynthesis-related genes/gene clusters, which might be relevant for carotenoid biosynthesis in this strain. Genes coding for different types of polyketide synthases (PKSs) or non-ribosomal peptide synthetases (NRPSs) were not identified in strain V22^T^. The same is true for clusters putatively involved in the synthesis of ectoine, bacteriocins and resorcinol (Table 2). *A. californiensis* CA12^T^ codes for an additional type III PKS compared to strain V22^T^, whereas a total number of 8 clusters are present in the genome of *P. piriforme* P3^T^. In conclusion, strain V22^T^ either produces only a very small set of secondary metabolites or harbours as yet uncharacterised clusters which escaped the bioinformatic prediction.

**Table 2:** Genome-based analysis of carbohydrate-active enzymes and secondary metabolite-related gene clusters

Feature	V22^T^	*A. californiensis* CA12^T^	*P. piriforme* P3^T^
Genome size (Mb)	5.16	5.48	6.32
Carbohydrate-active enzymes			
Glycoside hydrolase family	23	28	n.d.
Glycosyl transferase family	53	71	n.d.
Polysaccharide lyase family	2	6	n.d.
Carbohydrate esterase family	10	11	n.d.
Carbohydrate-binding module family	8	6	n.d.
Total	96	122	n.d.
Secondary metabolite-related gene clusters			
Terpenoid	3	3	4
Type I PKS	0	0	1
Type II PKS	0	0	0
Type III PKS	0	1	1
NRPS	0	0	0
Bacteriocin	0	0	2
Ectoine	0	0	0
Resorcinol	0	0	0
Total	3	4	8

The genome analysis is based on GenBank accession numbers CP036316 (V22^T^), CP036265 (*Alienimonas californiensis* CA12^T^) and GCA_900113665 (*Planctomicrobium piriforme* P3^T^); *n.d.* not determined

Taken together, the observed differences during comparison of morphological and genomic features of strain V22^T^ and its neighbours support the results of the phylogenetic analysis, which delineate strain V22^T^ from members of the genera *Alienimonas* and *Planctomicrobium*. We thus conclude that strain V22^T^ should be classified as the type strain of a novel species within a novel genus, for which we propose the name *Calycomorphotria* gen. nov., with the type species *Calycomorphotria hydatis* sp. nov.

### *Calycomorphotria* gen. nov.

Ca.ly.co.mor.pho’tri.a. Gr. n. *kalyx* a bud; Gr. fem. n. *morphotria* a creator; N.L. fem. n. *Calycomorphotria*, a bacterium that generates buds.

Members of the genus have a Gram-negative cell envelope architecture, are motile aerobic heterotrophs with a mesophilic, neutrophilic or slightly alkaliphilic growth profile. Cells are round grain rice-shaped, divide by budding and contain fimbriae and crateriform structures. The genus belongs to the family *Planctomycetaceae*, order *Planctomycetales*, class *Planctomycetia*, phylum *Planctomycetes*. The type species of this genus is *Calycomorphotria hydatis.*

### *Calycomorphotria hydatis* sp. nov.

hy’da.tis. Gr. n. *hydor*, -*atos* water; N.L. gen. n. *hydatis* of water; corresponding to the origin of the type strain from an aquatic environment.

Cells have an average size of 1.6 ± 0.3 × 0.7 ± 0.2 µm and form rosettes typically consisting of 6–15 cells. Rosettes assemble into linear or slightly branched aggregates. Cells contain crateriform structures covering the entire cell surface and a short stalk opposite of the budding pole. The cell plan features a condensed nucleoid, invaginations of the cytoplasmic membrane and cytoskeletal elements in the cytoplasm. Colonies are pink. Grows at ranges of 15–32 °C (optimum 30 °C) and at pH 6.0–10.0 (optimum 8.5). The G + C content of the type strain is 53.9%.

The type strain, V22^T^ (DSM 29767^T^ = LMG 29080^T^), was isolated from water and sediment of a seawater fish tank in June 2013. The type strain genome (acc. no. CP036316) and 16S rRNA gene sequence (acc. no. MK554537) are available from GenBank. The genome size of the type strain is 5,163,473 bp.
